# A Novel Type of Influenza A Virus-Derived Defective Interfering Particle with Nucleotide Substitutions in Its Genome

**DOI:** 10.1128/JVI.01786-18

**Published:** 2019-02-05

**Authors:** Sascha Young Kupke, Dietmar Riedel, Timo Frensing, Pawel Zmora, Udo Reichl

**Affiliations:** a Max Planck Institute for Dynamics of Complex Technical Systems, Department of Bioprocess Engineering, Magdeburg, Germany; b Max Planck Institute for Biophysical Chemistry, Facility for Transmission Electron Microscopy, Göttingen, Germany; c Otto von Guericke University Magdeburg, Chair of Bioprocess Engineering, Magdeburg, Germany; Icahn School of Medicine at Mount Sinai

**Keywords:** defective interfering particles, influenza A virus, single-cell analysis

## Abstract

Defective interfering particles (DIPs) replicate at the expense of coinfecting, fully infectious homologous virus. Typically, they contain a highly deleted form of the viral genome. Utilizing single-cell analysis, here we report the discovery of a yet-unknown DIP type, derived from influenza A viruses (IAVs), termed OP7 virus. Instead of deletions, the genomic viral RNA (vRNA) of segment 7 (S7) carried 37 point mutations compared to the reference sequence, affecting promoter regions, encoded proteins, and genome packaging signals. Coinfection experiments demonstrated strong interference of OP7 virus with IAV replication, manifested by a dramatic decrease in the infectivity of released virions. Moreover, an overproportional quantity of S7 in relation to other genome segments was observed, both intracellularly and in the released virus population. Concurrently, OP7 virions lacked a large fraction of other vRNA segments, which appears to constitute its defect in virus replication. OP7 virus might serve as a promising candidate for antiviral therapy. Furthermore, this novel form of DIP may also be present in other IAV preparations.

**IMPORTANCE** Defective interfering particles (DIPs) typically contain a highly deleted form of the viral genome, rendering them defective in virus replication. Yet upon complementation through coinfection with fully infectious standard virus (STV), interference with the viral life cycle can be observed, leading to suppressed STV replication and the release of mainly noninfectious DIPs. Interestingly, recent research indicates that DIPs may serve as an antiviral agent. Here we report the discovery of a yet-unknown type of influenza A virus-derived DIP (termed “OP7” virus) that contains numerous point mutations instead of large deletions in its genome. Furthermore, the underlying principles that render OP7 virions interfering and apparently defective seem to differ from those of conventional DIPs. In conclusion, we believe that OP7 virus might be a promising candidate for antiviral therapy. Moreover, it exerts strong effects, both on virus replication and on the host cell response, and may have been overlooked in other IAV preparations.

## Abstract (precis)

Defective interfering particles (DIPs) typically contain a highly deleted form of the viral genome, rendering them defective in virus replication. Yet upon complementation through coinfection with fully infectious standard virus (STV), interference with the viral life cycle can be observed, leading to suppressed STV replication and the release of mainly noninfectious DIPs. Interestingly, recent research indicates that DIPs may serve as an antiviral agent. Here we report the discovery of a yet-unknown type of influenza A virus-derived DIP (termed “OP7” virus) that contains numerous point mutations instead of large deletions in its genome. Furthermore, the underlying principles that render OP7 virions interfering and apparently defective seem to differ from those of conventional DIPs. In conclusion, we believe that OP7 virus might be a promising candidate for antiviral therapy. Moreover, it exerts strong effects, both on virus replication and on the host cell response, and may have been overlooked in other IAV preparations.

## INTRODUCTION

Defective interfering (DI) particles (DIPs) are of viral origin and share the same structural features as their homologous standard viruses (STVs), yet they typically contain a heavily deleted form of the viral genome (1). As a result of the missing genomic information, DIPs are defective in virus replication and, hence, cannot result in the production of progeny virions, once infecting a cell. However, upon complementation by coinfection with a fully infectious STV, interference with the normal viral life cycle can be observed, with suppressed STV replication and the release of mainly noninfectious DIPs. This infection outcome is a result of the growth advantage of the DI genome over the full-length (FL) counterpart, which is manifested by enhanced genomic replication, outcompetition for cellular or viral resources, and preferential packaging into virus particles (2). Furthermore, considering the ability of DIPs to suppress virus replication, a growing interest in the potential application of DIPs as an antiviral agent can be observed (3–6).

DIPs were observed for most DNA and RNA viruses, including viruses containing single- and double-stranded genomes. The internal genomic deletions are suggested to arise by erroneous translocation of the viral polymerase during genomic replication, often referred to as the “copy choice” mechanism (7, 8). Other DI genomes include multiple deleted forms, “copyback” or “hairpin” genomes (some parts are repeated in the reverse complement form), and “mosaic” genomes (multiple nonadjacent sections are joined together). The precise mechanisms of interference are not yet fully understood. However, it was suggested that DI genomes compete for helper virus-encoded gene products (7, 8), in particular for viral polymerases (9, 10). Furthermore, for influenza A viruses (IAVs), a preferential synthesis of the DI genome over the FL counterpart was observed (11, 12). In this context, it was proposed that DI genomes show faster accumulation, due to their reduced length (2, 13, 14). Moreover, DI genomes of IAVs competitively inhibited the packaging of, specifically, their FL parental genomic viral RNA (vRNA) and were further preferentially incorporated into progeny virions (12, 15).

So far, DI genomes have been primarily identified based on their large genomic deletions, and only little attention was paid to potential nucleotide substitutions. Utilizing single-cell analysis, here we report the discovery of a novel type of IAV-derived DIP, which carries numerous point mutations in one of its eight genomic negative-sense RNA segments. Recently, using single-cell analysis, we revealed a large cell-to-cell heterogeneity in IAV replication, with an almost 1,000-fold difference in virus titers and intracellular vRNA levels (16). Interestingly, between single cells, the different genome segments showed a positive correlation in their quantities, except for segment 7 (S7) vRNA, which was not correlated with any other segment. Yet so far, no valid explanation could be provided for this phenomenon.

In the present study, we followed up on our previous single-cell infection experiments using IAV (16) and observed a subset of single cells that showed an unusual phenotype, characterized by a low infectious titer of the viral progeny and an overproportional intracellular quantity of S7 vRNA in relation to other genome segments. We show that this was caused by coinfection of a subpopulation of viruses, termed overproportional S7 (OP7) virus. Following its enrichment, we determined the sequence of the genomic vRNA of S7 from OP7 virions (S7-OP7) that shows 37 point mutations in relation to the reference sequence, affecting promoter regions, encoded proteins, and genome packaging signals. Furthermore, cell population-based infection experiments with OP7 seed viruses showed that (i) the altered viral RNA synthesis can be accounted for by the promoter mutation identified on S7-OP7 and (ii) the released OP7 virions appear to be defective in virus replication due their incomplete vRNA content, except for S7-OP7, which was predominantly packaged. Finally, coinfection experiments demonstrated a strong interference of OP7 virus with the replication of relevant IAV strains, and interference in human cell lines, which may render them promising for utilization as an antiviral agent. Moreover, our results unveil that OP7 virions are a yet-unidentified form of DIP, derived from IAVs, with point mutations instead of deletions in its genome.

## RESULTS

### Single-cell analysis indicates the presence of a viral subpopulation with an unusual phenotype in PR8 virus.

**FIG 1:**
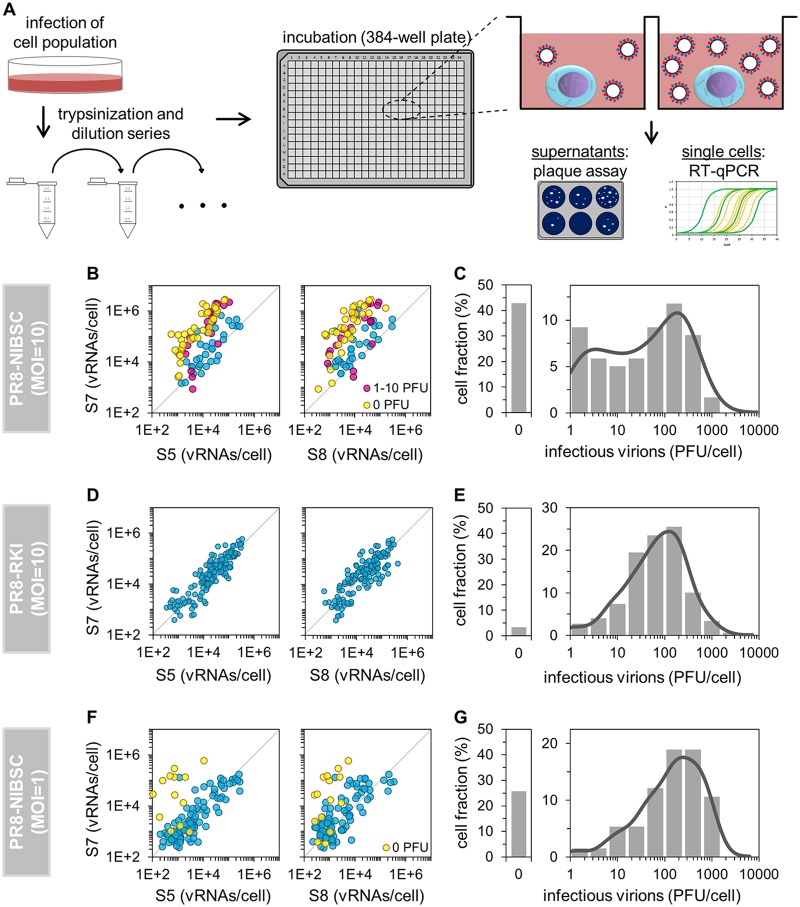
Single-cell analysis workflow and dependence of virus titers on the S7 vRNA level. (A) Scheme of the experimental approach. IAV-infected MDCK cells were trypsinized, serially diluted, and transferred to a 384-well plate. Wells containing single cells were identified by microscopy. At 12 hpi, virus titers in the supernatant were quantified by a plaque assay, and intracellular vRNAs were quantified by real-time RT-qPCR. (Scheme adapted from reference 16.) (B, D, and F) Effect of the vRNA level on virus yield. Colors highlight virus release, whenever significant; otherwise, cells are blue. The parity line (*r* = 1) is shown for reference. (C, E, and G) Distributions of virus titers. Solid lines indicate the probability density function (calculated by Kernel density estimation). Cells that were tested negative in both intracellular vRNAs and released PFU (noninfected cells) were excluded from the analysis of infections performed at an MOI of 1 (F and G). Illustrations include pooled data from multiple independent experiments (*n* = 4 for panels B and C, yielding 119 cells; *n* = 4 for panels D and E, yielding 149 cells; and *n* = 3 for panels F and G, yielding 132 cells).

To study the dependence of virus release on the intracellular S7 vRNA quantity, which showed large cell-to-cell variability (16), we performed single-cell analysis of infected cells (Fig. 1A). A population of adherent Madin-Darby canine kidney (MDCK) cells was infected with IAV and then trypsinized to obtain a cell suspension. The diluted cell suspension was transferred to a 384-well plate to obtain (on average) 1 cell per well, and wells containing single cells were identified by microscopy. At 12 h postinfection (hpi), we quantified virus titers from these cells using a plaque assay. In addition, cells were lysed and analyzed for intracellular vRNAs by real-time reverse transcription-quantitative PCR (RT-qPCR). Infection experiments here were performed with the influenza virus A/Puerto Rico/8/34 (PR8) strain from the National Institute for Biological Standards and Control (PR8-NIBSC) or from the Robert Koch Institut (PR8-RKI).

**FIG 2:**
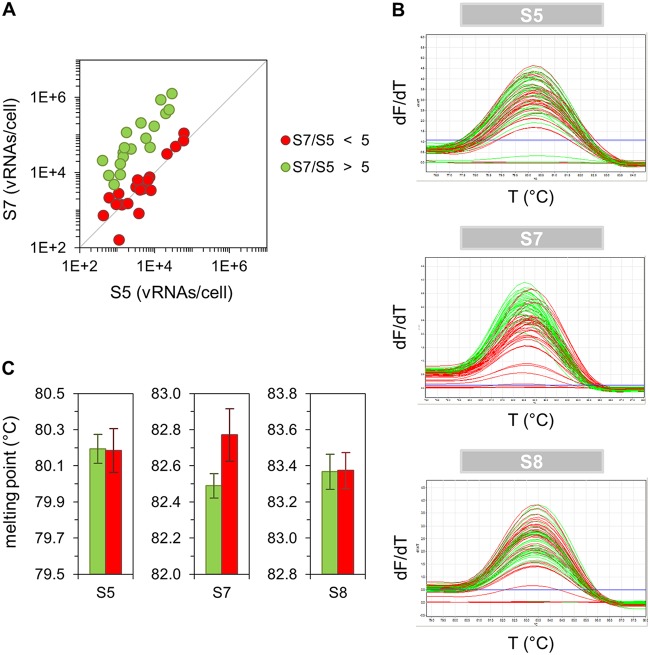
Melting-curve analysis of qPCR amplicons. Infected single MDCK cells (derived from a cell population infected with PR8-NIBSC at an MOI of 10, as described above [Fig. 1A]) were cultivated until 12 hpi and subsequently assayed for their intracellular vRNAs by real-time RT-qPCR. Subsequent to qPCR, melting-curve analysis was performed. (A) Correlation between vRNA segments. Cells with equimolar and overproportional levels of S7 (compared to S5) are shown in red and green, respectively. (B) Melting curves of qPCR amplicons. T, temperature; dF/dT, change in fluorescence divided by change in temperature. (C) Comparison of melting points. Error bars indicate standard deviations of the mean values depicted. The result of one representative experiment is shown, yielding 38 cells.

Surprisingly, upon infection with PR8-NIBSC at a multiplicity of infection (MOI) of 10, individual cells that showed a low infectious virus titer (0 to 10 PFU) contained a relatively high and disproportionate level of S7 vRNA in relation to S5 or S8 (Fig. 1B). In particular, cells showing no plaque titer (0 PFU) almost exclusively contained this overproportional quantity of S7 vRNA. Most of the cells that released 1 to 10 PFU contained such levels as well. Furthermore, the distribution of virus titers between single cells appeared to be bimodal, as two subpopulations of cells could be observed, including a subset that released about 1 to 10 PFU (Fig. 1C). In addition, it seemed that cells with overproportional S7 levels contained a different S7 vRNA sequence (compared to cells with equimolar ratios), as indicated by the different denaturation temperatures of S7 amplicons in a melting-curve analysis (Fig. 2). We thus hypothesized that PR8-NIBSC may contain a subpopulation of virions with a different S7 segment.

To test whether such a subpopulation was also present in a different seed virus, we infected cells with PR8-RKI at an MOI of 10. However, no such unusual behavior was observed for S7. We did not observe overproportional levels of S7 vRNA in comparison to S5 or S8 (Fig. 1D), nor did we recognize any bimodality in the histogram of virus titers (Fig. 1E). Concurrently, the fraction of cells showing no virus release was very small for PR8-RKI virus replication (only 3% compared to 43% for infection with PR8-NIBSC virus).

Interestingly, the occurrence of the unusual phenotype was reduced upon infection with PR8-NIBSC at an MOI of 1. More specifically, fewer cells contained an overproportional level of S7 vRNA (Fig. 1F) than in infections performed at the higher MOI (Fig. 1B). Moreover, the fraction of cells showing no virus release was decreased (26% in comparison to 43%), and a bimodal distribution of virus titers was no longer apparent (Fig. 1G). Hence, we presumed that replication of the putative subpopulation of virus in PR8-NIBSC may depend on coinfection with STVs, which are less frequent at an MOI of 1. Furthermore, we concluded that coinfection with such viruses results in an unusual phenotype, characterized by a low infectious virus titer and an overproportional intracellular level of S7 vRNA in relation to other genome segments.

### The OP7 virus subpopulation can be enriched using single-cell infection experiments and depleted by plaque purification.

**FIG 3:**
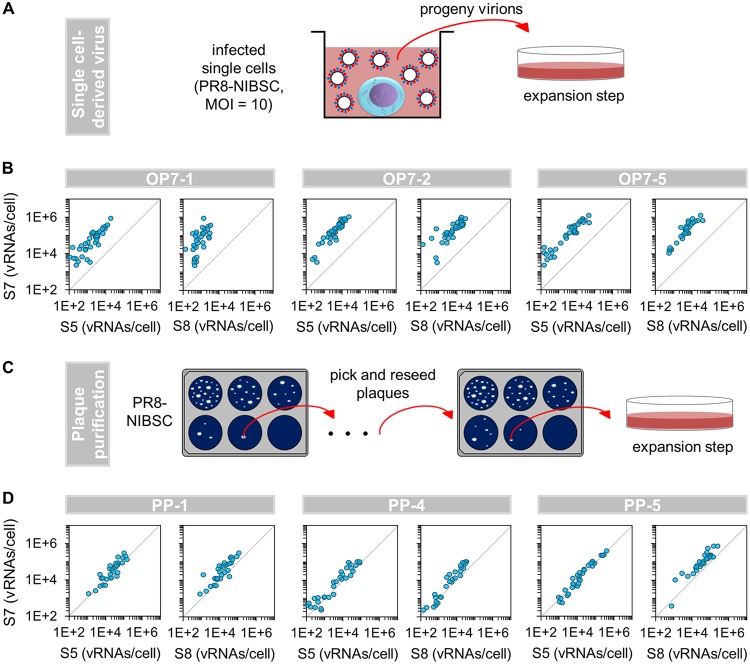
Enrichment of OP7 virus. (A) Generation of single-cell-derived virus seeds. Infected single MDCK cells (derived from a cell population infected with PR8-NIBSC at an MOI of 10, as described above [Fig. 1A]) were cultivated until 12 hpi. Subsequently, whole supernatants (containing all progeny virions) were expanded in confluent MDCK cells. Four independent experiments resulted in 55 virus seeds, of which five isolates showed a strongly pronounced OP7 virus phenotype (panel B and Fig. 4). (Adapted from reference 16.) (B and D) Correlation between vRNA segments in infected single MDCK cells. Selected OP7 (B) and PP (D) seed viruses (preparation shown in panel A and in panel C, respectively) were used to infect MDCK cells at an MOI of 10. Single cells were then isolated as described above (Fig. 1A). At 12 hpi, cells were assayed for vRNAs via real-time RT-qPCR. Independent experiments were conducted, each using one virus seed, yielding 31 to 40 single-cell measurements each. The parity line (*r* = 1) is shown for reference. (C) Scheme of the plaque purification procedure. Plaques from PR8-NIBSC virus were picked and reseeded in three consecutive assays and finally propagated in confluent MDCK cells. Two independent experiments yielded 43 PP virus isolates.

To investigate whether we can enrich the putative viral subpopulation in the PR8-NIBSC seed virus, we performed single-cell infection experiments at an MOI of 10 (as described above [Fig. 1A]), and progeny virions in the complete supernatants of individual cells were expanded using confluent MDCK cells (Fig. 3A) to yield seed viruses. Second, to test whether the putative subpopulation of virus can be depleted by the exclusion of coinfection events, we utilized plaque purification. For this, we picked and reseeded individual plaques from PR8-NIBSC virus in three consecutive assays (Fig. 3C). The obtained virus was then multiplied in MDCK cells to yield virus seeds. All seed viruses obtained were then titrated for subsequent single-cell experiments at an MOI of 10, as described above (Fig 1A).

**FIG 4:**
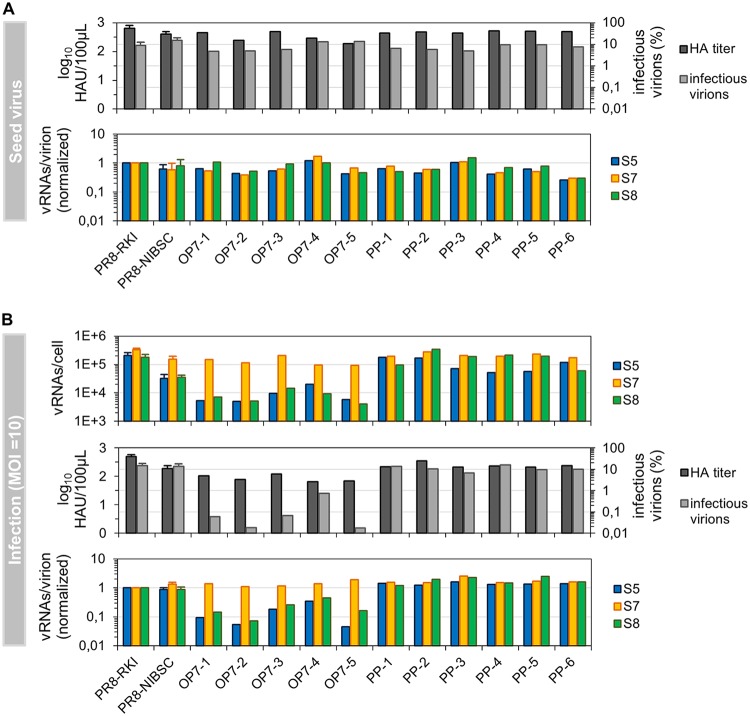
Cell population-based infections with OP7 seed viruses. (A) Infectivity and vRNA content of OP7 and PP seed viruses (from Fig. 3A and C, respectively). Infectious virus titers were quantified by a TCID_50_ assay, and purified vRNAs from virions were quantified by real-time RT-qPCR. Data were used to calculate fractions of infectious virus and numbers of vRNAs per virion based on the virus particle concentration (derived from the HA titer). Normalization of vRNAs per virion was based on PR8-RKI virus (as a reference). (B) Outcome of high-MOI experiments using the seed viruses shown in panel A. MDCK cells, infected at an MOI of 10, were assayed for the per-cell vRNA content at 12 hpi. Infectivity and vRNAs per virion are given for produced virions. Infection experiments with PR8-RKI and PR8-NIBSC viruses were performed in independent experiments (*n* = 3) and once with each OP7 and PP seed virus. Error bars indicate standard deviations of depicted mean values.

Indeed, infection experiments with three selected single-cell-derived virus seeds (of 55 isolates) showed a strongly pronounced, unusual phenotype (Fig. 3B). In particular, the infected cells exclusively contained an overproportional level of S7 vRNA in relation to S5 or S8. These viruses are referred to here as “OP7 seed virus.” Moreover, 93% of cells infected with OP7 seed virus 1 (OP7-1) showed no virus release, while for OP7-2 and OP7-5, the fractions were 95% and 90%, respectively. The remaining cells produced very low virus titers (1 to 10 PFU). Note that only 5 of the 55 single-cell-derived virus seeds (obtained in four independent experiments) showed a strong form of the unusual phenotype (all five OP7 seed viruses are shown in Fig. 4), and an additional ∼20% of the isolates showed a weak phenotype and were excluded from further experiments.

In addition, none of the cells infected with plaque-purified (PP) virus showed the above-mentioned unusual phenotype (Fig. 3D). More precisely, an overproportional quantity of intracellular S7 vRNA (compared to S5 and S8) was not observed. Furthermore, only 4% of cells showed no virus release upon infection with PP virus 1 (PP-1). Upon PP-4 and PP-5 virus infection, these fractions were 0% and 5%, respectively. In total, 43 plaque-purified viruses were generated (in two independent experiments), and all isolates were tested negative regarding the unusual phenotype (selected isolates are shown in Fig. 4). Note that due to the limited volumes (∼2 ml) of OP7 and PP seed viruses generated, only a low number of aliquots could be prepared, which allowed only a limited number of subsequent infection experiments. We thus used three different OP7 seed viruses, each in one independent infection, for each subsequent experiment. Note that in Fig. 4, all relevant isolates (investigated in this study) are shown. Taken together, our results demonstrate the presence of an OP7 virus subpopulation in the PR8-NIBSC seed virus and, furthermore, that it can be enriched utilizing single-cell infection experiments and depleted by plaque purification.

### OP7 virions appear to be noninfectious due to their incomplete vRNA content, except for S7, which predominates in the virions.

As OP7 virus was successfully enriched, we next performed cell population-based experiments to explore additional features of OP7 seed virus infection. For this, we used the OP7 and PP seed viruses produced as described above (Fig. 3A and C, respectively). MDCK cells were infected at an MOI of 10 and assessed for virus titers by a hemagglutination assay (HA) and for infectious virions by a 50% tissue culture infective dose (TCID_50_) assay at 12 hpi. Intracellular vRNAs and vRNAs of released virions were quantified by real-time RT-qPCR. Note that S5, S7, and S8 were quantified representatively for all genome segments.

Surprisingly, we did not find remarkable differences in the properties of OP7 seed viruses compared to PR8-RKI, PR8-NIBSC, and the PP viruses (Fig. 4A). All viruses showed high infectious titers, most likely due to the predominant presence of fully infectious STV. However, upon infection with OP7 seed virus at an MOI of 10, we again observed an overproportional quantity of intracellular S7 vRNA in relation to S5 and S8 (Fig. 4B, top panel), similarly to our previous single-cell experiments. Interestingly, the levels of S5 and S8 were significantly reduced compared to PR8-RKI and PP virus replication (by at least 1 order of magnitude).

**FIG 5:**
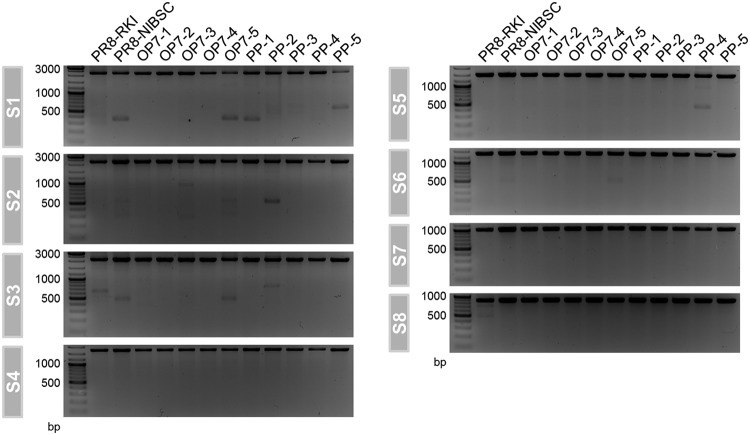
Subgenomic vRNAs in virus particles. Released viruses from MDCK cells, infected at an MOI of 10 (from the experiment shown in Fig. 4B), were investigated for the presence of subgenomic vRNAs on S1 to S8 by segment-specific RT-PCR at 12 hpi. FL and DI vRNAs appear in the top and bottom parts of the gel, respectively.

Moreover, the majority of virus progeny from OP7 seed virus-infected cells were noninfectious (Fig. 4B, middle panel). Specifically, in comparison to PR8-RKI or PP virus replication, we observed reductions in the infectivity of produced virions of almost 3 log_10_ units for OP7-5 and more than 1 log_10_ unit for OP7-4 seed virus infections. The HA titer upon OP7 seed virus infection was (on average) reduced by 0.8 log_10_ units compared to PR8-RKI and at least 0.3 log_10_ units lower than with PP virus replication. The low percentage of infectious virions cannot be explained by the presence of conventional DIPs, as the results of segment-specific PCR did not indicate a pronounced accumulation of subgenomic vRNAs in the produced virions upon OP7 seed virus infection (Fig. 5). Below, we refer to virus particles released in infections with OP7 seed viruses as “OP7 virions.”

**FIG 6:**
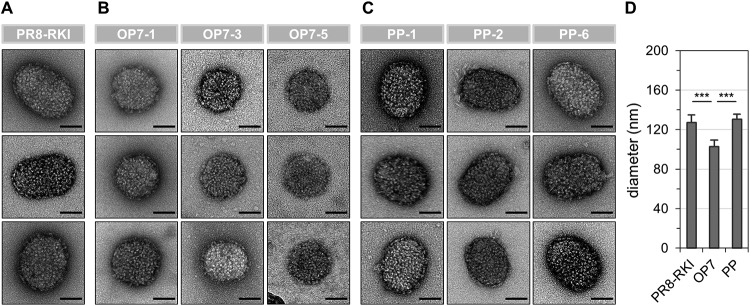
Virus particles imaged by ns-TEM. Released virions from infected MDCK cells (MOI = 10; 12 hpi) (experiment described in the legend of Fig. 4B) are shown. (A to C) Representative virus particles for PR8-RKI (A), OP7 (B), and PP (C) virus. Bars, 50 nm. (D) Diameters of virions determined from ns-TEM images. For nonspherical particles, we determined means of the length and width. Diameters of 16, 17, and 23 virions were determined for PR8-RKI, OP7, and PP viruses, respectively. Error bars indicate standard deviations. ***, *P* < 0.001 by Student’s *t* test.

The low infectivity of OP7 virions can rather be explained by their low vRNA content (Fig. 4B, bottom panel). More specifically, the calculated numbers of S5 and S8 vRNAs per virion were reduced by approximately 1 order of magnitude compared PR8-RKI and PP virus particles. Intriguingly, the number of S7 vRNAs was not affected. Hence, this result clearly indicates that OP7 virions are incomplete with respect to their vRNA content (except for S7), which would render them unable to reproduce upon a single-hit infection. The remaining infectivity is most likely conferred by the presence of STV. Furthermore, OP7 virions were smaller than PR8-RKI and PP virions, as indicated by negative-stain transmission electron microscopy (ns-TEM) (Fig. 6). However, particle morphology did not seem to be affected, as we observed spherical OP7 virus particles with well-resolved surface spike proteins. In summary, our data strongly suggest that OP7 virions are noninfectious as a result of their lack of genomic vRNA content, with the exception of S7, which was predominantly incorporated.

### Nucleotide substitutions in vRNA of S7-OP7 affecting encoded proteins, packaging signals, and promoter regions.

**FIG 7:**
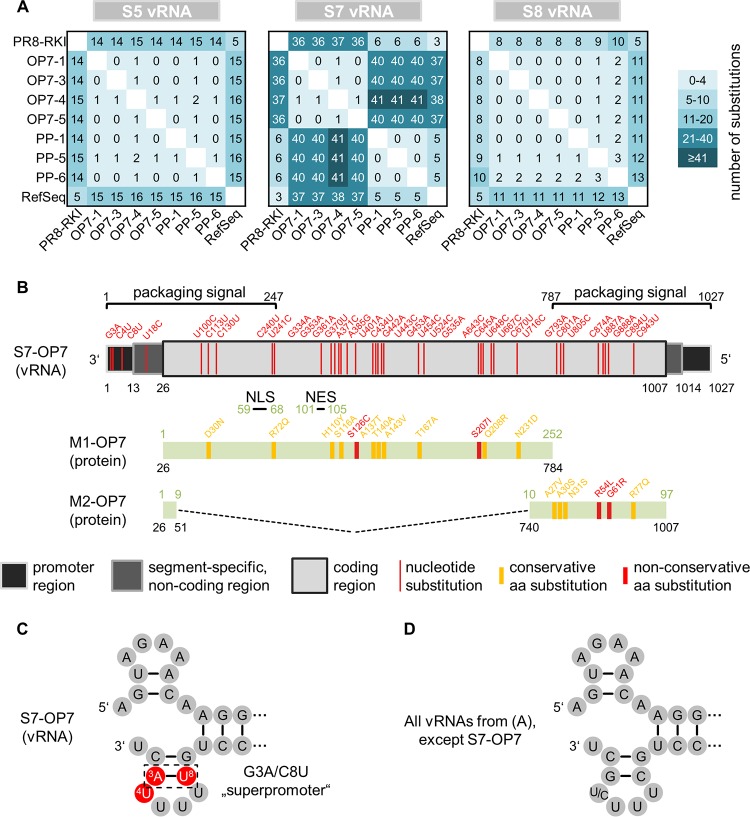
Nucleotide substitutions in genomic S7-OP7 vRNA. (A) Comparison of vRNA sequences. Sequences were determined from virions released at a high MOI (from experiments depicted in Fig. 4B). (B) Alterations in the functional regions of S7-OP7 vRNA. Nucleotide and amino acid (aa) positions are indicated in black and green numbers, respectively. (C and D) Corkscrew structures adopted by promoter regions. GenBank accession numbers of all vRNA sequences are provided in Materials and Methods (see “Data availability”).

Next, we determined the sequences of vRNAs from OP7 virions to elucidate whether they contain genomic mutations. Our experiments revealed a significant number of point mutations on the vRNA of S7-OP7 (Fig. 7A). The number of substitutions ranged from 36 to 41 in comparison to PR8-RKI, PP virus, and the reference sequence (RefSeq) of PR8 from the National Center for Biotechnology Information (NCBI) (accession number NC_002016.1). In contrast, S5 and S8 showed fewer alterations, with substitutions in 8 to 16 nucleotides (nt) compared to PR8-RKI virus and the NCBI RefSeq (accession numbers NC_002019.1 for S5 and NC_002020.1 for S8) and in 0 to 3 nt in comparison to PP virus. GenBank accession numbers of all vRNA sequences determined are given in Materials and Methods (see “Data availability,” below).

**FIG 8:**
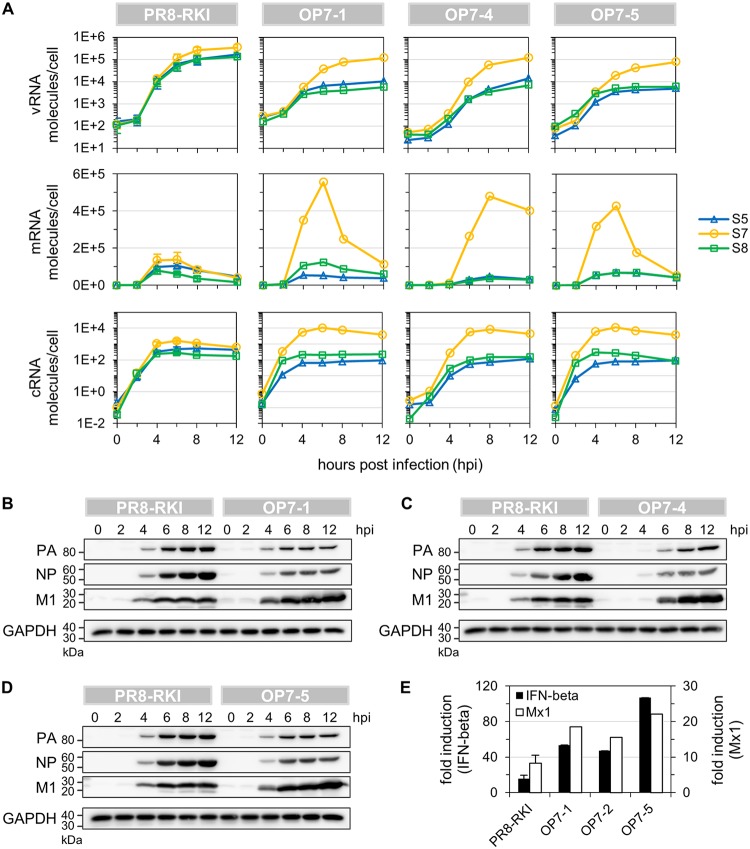
Viral RNA synthesis in OP7 seed virus-infected cells. MDCK cells infected at an MOI of 10 were assayed for intracellular viral RNAs by real-time RT-qPCR and for viral protein content by WB. (A) Intracellular dynamics of vRNA, mRNA, and cRNA quantities. (B to D) WB analysis of intracellular viral protein accumulation. (E) Induction of the innate immune response at 12 hpi. IFN beta and Mx1 expression levels were measured by real-time RT-qPCR and expressed as fold induction (over mock-infected cells) using the ΔΔ*C_T_* method. Infections with PR8-RKI were performed in independent experiments (*n* = 3) and once with each OP7 seed virus. Error bars indicate standard deviations of the mean values depicted.

**FIG 9:**
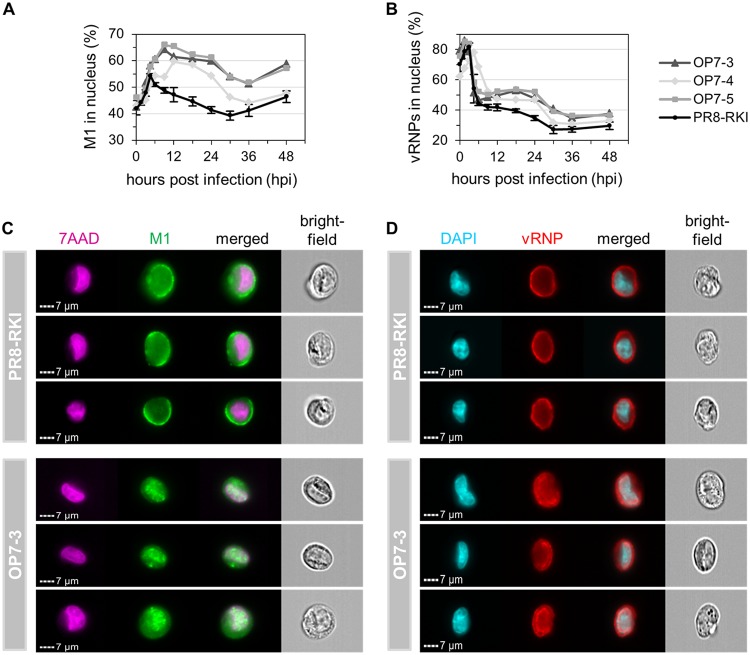
Intracellular M1 and vRNP localization dynamics upon OP7 seed virus infection. Imaging flow cytometric analysis was performed for infected MDCK cells (MOI = 10). (A and B) Dynamics of nuclear localization of M1 (A) and vRNPs (B). Cells were stained for either M1 or vRNPs and cell nuclei using 7-AAD or DAPI, respectively. Fractions of M1 or vRNPs in the nucleus were calculated based on the amount of fluorescence signal colocalized with the nuclear signal. A total of 10,000 single cells were evaluated per sample. Infections with PR8-RKI virus were performed in independent experiments (*n* = 3) and once with each OP7 seed virus. Error bars indicate standard deviations of illustrated mean values. Statistical analysis of data depicted in panel B is shown in Fig. 10. (C and D) Images of representative cells stained for M1 at 9 hpi (C) and for vRNPs at 18 hpi (D). Panels for one representative experiment are depicted.

Figure 7B illustrates the 37 point mutations of S7-OP7 vRNA in relation to the RefSeq, which concern several functional regions of the genome segment. Note that the investigated OP7 virus isolates showed an identical S7 sequence, except for OP7-4, which showed an additional substitution. This isolate was excluded from the analysis, as the OP7 phenotype was overall less pronounced (Fig. 4, 8, 9, and 11). The coding region contains 33 point mutations, resulting in 10 conservative and 2 nonconservative amino acid substitutions for the encoded matrix protein 1 (M1) and 4 conservative and 2 nonconservative substitutions for matrix protein 2 (M2). The M1 nuclear localization signal (NLS) (17) and nuclear export signal (NES) (18) did not show alterations, and no additional stop codons were observed in the M1 and M2 reading frames. Moreover, we did not find mutations at sites that affect splicing of M2 mRNA.

Four nucleotide substitutions were observed in the untranslated regions (UTRs), which involve the promoter regions and segment-specific noncoding regions (NCRs) at both vRNA ends. The promoter regions are highly conserved and comprise the noncoding 13 and 12 nt at the 5′ and 3′ ends of vRNA, respectively (19). Yet on S7-OP7 vRNA, we identified the G3A/C8U substitutions (Fig. 7B and C), which were previously described to result in the formation of the so-called “superpromoter” (20). Furthermore, we identified a substitution at the fourth position (C4U) at the 3′ end, which is usually polymorphic (U/C), depending on the genome segment (21). Nucleotide substitutions at these three positions were found neither on other segments of OP7 virions nor on all segments of PP and PR8-RKI viruses (Fig. 7D). Furthermore, the segment-specific genome packaging signal sequences of S7 (22), which include the UTRs and proximal parts of the coding region at both vRNA ends, were affected by 17 point mutations (Fig. 7B). Taken together, the vRNA of S7-OP7 shows a significant amount of nucleotide substitutions, while the extent of substitutions in S5 and S8 sequences is lower. The 37 point mutations were distributed in the entire genome segment, affecting the M1 and M2 protein sequences, promoter regions, and genome packaging signals.

### Strongly altered intracellular viral RNA dynamics upon OP7 seed virus infection.

Each genomic vRNA segment is encapsidated into a viral ribonucleoprotein (vRNP) complex, involving viral nucleoproteins (NPs) and the tripartite viral polymerase (23). Once in the nucleus, they are engaged in both transcription of viral mRNA and replication of cRNA. cRNAs are themselves encapsidated in cRNPs and serve as a replication intermediate for the synthesis of progeny vRNA (24). To study the potential effect of the promoter mutations (found on the vRNA of S7-OP7) on viral RNA synthesis upon OP7 seed virus infection, we next investigated intracellular viral RNAs by real-time RT-qPCR and viral proteins by Western blotting (WB). Below, we use PR8-RKI (and not PR8-NIBSC) virus for the “reference” or “PR8 wild-type” (WT) virus infection, as we show that (i) OP7 virions are present in the PR8-NIBSC virus seed (Fig. 1 and 3), (ii) they seem to influence PR8-NIBSC virus replication (Fig. 1 and 4), and (iii) PR8-RKI seed virus appeared to be devoid of OP7 virions (Fig. 1, 4, and 7).

Until 12 hpi, the vRNA of S7 in OP7 seed virus infection reached quantities that were comparable to the levels of S5, S7, and S8 in PR8-RKI virus replication (Fig. 8A). However, the levels of S5 and S8 vRNAs in OP7 seed virus infection were significantly reduced (by approximately 1 order of magnitude) in relation to S7, in agreement with our above-described observation (Fig. 4B). The mRNA of S7 reached high peak levels compared to S5 and S8 and in relation to all mRNAs of PR8-RKI virus replication, with a 3- to 6-fold increase observed between 6 and 8 hpi. Similarly, S7 cRNA reached elevated levels upon OP7 seed virus infection in comparison to other segments’ cRNAs and compared to all measured cRNAs of PR8-RKI virus replication. This increase was roughly 7-fold between 6 and 8 hpi in relation to PR8-RKI virus replication. The quantity of S8 cRNA in OP7 seed virus infection was comparable to that in PR8-RKI virus replication; however, the level of S5 cRNA was slightly reduced.

In addition, intracellular M1 protein appeared to accumulate to high levels upon OP7 seed virus infection in comparison to PR8-RKI virus replication, while the amounts of the NP and polymerase acid (PA) proteins seemed to be reduced (Fig. 8B to D). Furthermore, we observed enhanced type I interferon (IFN) induction in OP7 seed virus-infected cells compared to PR8 RKI virus replication, as indicated by elevated IFN beta and myxovirus-resistant gene 1 (Mx1) transcript levels (Fig. 8E). In summary, compared to WT virus infection, strongly altered intracellular viral RNA dynamics can be observed upon OP7 seed virus infection.

### Enhanced nuclear accumulation of mutated M1-OP7 may cause nuclear retainment of vRNPs.

Once in the nucleus, M1 mediates the nuclear export of vRNPs (25). As the M1 protein of OP7 virus (M1-OP7) showed modifications, we next explored whether intracellular protein trafficking was altered upon OP7 seed virus infection. To this end, we used imaging flow cytometry. Infected cells were stained using either anti-M1 or anti-vRNP monoclonal antibodies (mAbs) in combination with the nuclear stain 7-aminoactinomycin D (7-AAD) or 4′,6-diamidino-2-phenylindole (DAPI), respectively. Fractions of the respective proteins/complexes in the cell nuclei were calculated based on the amount of the fluorescence signal that was colocalized with the nuclear signal.

**FIG 10:**
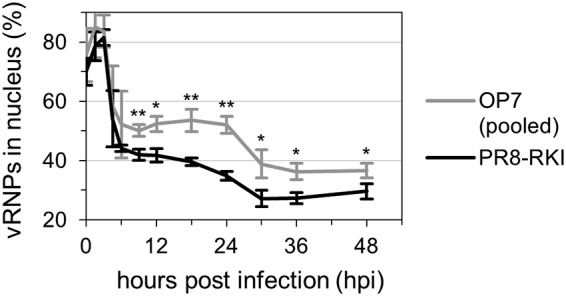
Statistical analysis of nuclear vRNP localization dynamics. Imaging flow cytometric analysis was performed for infected MDCK cells (MOI = 10). Cells were stained for vRNPs and cell nuclei. Fractions of vRNPs in the nucleus were calculated based on the amount of fluorescence signal colocalized with the nuclear signal. A total of 10,000 single cells were evaluated per sample. Infections with PR8-RKI virus were performed in independent experiments (*n* = 3) and once with each OP7 seed virus (i.e., OP7-3, OP7-4, and OP7-5 viruses) (see also Fig. 9). The data set for OP7 seed virus infections was pooled for analysis. Error bars indicate standard deviations of the means depicted. *, *P* < 0.05; **, *P* < 0.01 (by Student’s *t* test).

Until 4.5 hpi, the fraction of M1 in the nucleus was steadily increasing in PR8-RKI virus replication, indicating nuclear import subsequent to their production (Fig. 9A). Concurrently, from 3 to 4.5 hpi, the percentage of vRNPs in the nucleus shows a steep decrease, which indicates nuclear export of the viral genomes (Fig. 9B). Hence, the accumulation of M1 in the nucleus coincided with the nuclear export of vRNPs. In contrast, for OP7 seed virus-infected cells, we can observe a strong increase in the percentage of M1 in the nucleus even after 4.5 hpi (Fig. 9A), also illustrated by images shown in Fig. 9C. In addition, while a large proportion of vRNPs appeared to leave the nucleus from 3 to 4.5 hpi, some vRNPs seemed to remain in the nucleus from 9 hpi onwards, as indicated in comparison to PR8-RKI virus replication (Fig. 9B). This difference in the vRNP localization dynamics may appear less obvious than the difference in the localization dynamics of M1 (Fig. 9A). We therefore pooled the data from the three independent OP7 seed virus infection experiments (using a different OP7 seed virus in each experiment) for statistical analysis (Fig. 10), which demonstrated a significant difference in the nuclear vRNP localization dynamics in relation to PR8-RKI replication as well. Moreover, the nuclear retainment of vRNPs in OP7 virus replication is further visualized by the imagery shown in Fig. 9D. Note that due to the G3A/C8U superpromoter identified on the vRNA of S7-OP7, it has to be assumed that the majority of the synthesized M1 protein is likely M1-OP7. In summary, image flow cytometric analysis indicates an enhanced nuclear accumulation of the mutated M1-OP7 upon OP7 seed virus infection, which may cause the apparent nuclear retainment of a fraction of vRNPs.

### OP7 virus interferes with replication of IAVs in coinfection studies.

Conventional DI RNAs are thought to have growth advantages over their FL counterparts, i.e., enhanced genomic replication and preferential incorporation into progeny virions. Intriguingly, the mutated vRNA of S7-OP7 seemed to have very similar advantages in propagation. We therefore hypothesized that OP7 virus may even share another feature with conventional DIPs: interference with replication of STVs. To further explore this possibility, we simultaneously coinfected cells with IAV and OP7 virus.

**FIG 11:**
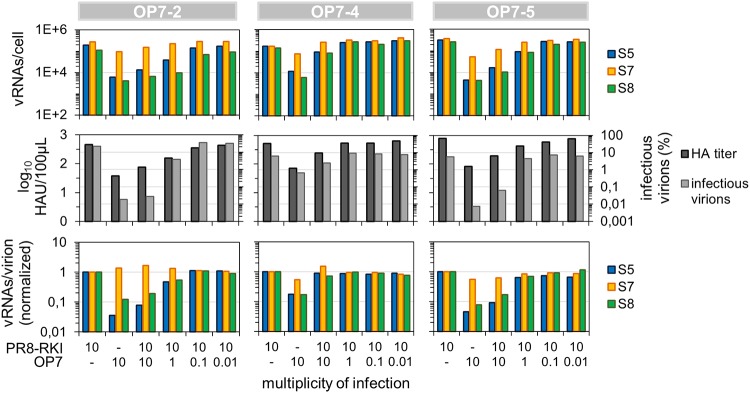
Coinfection of PR8-RKI virus-infected MDCK cells with OP7 seed virus. MDCK cells infected with PR8-RKI virus at an MOI of 10 were simultaneously coinfected with OP7 seed virus at the indicated MOIs until 12 hpi. Infectious virus titers were quantified by a plaque assay, and intracellular and purified vRNAs from virions were quantified by real-time RT-qPCR. Data were used to calculate fractions of infectious virus and numbers of vRNAs per virion using the virus particle concentration derived from the HA titer. Normalization of vRNAs per virion was based on PR8-RKI virus (as a reference). Three independent infection experiments were conducted, each using PR8-RKI and one OP7 seed virus.

Indeed, the coinfection experiments showed attenuated replication of PR8-RKI virus (Fig. 11). In comparison to cells infected with only PR8-RKI (MOI = 10), the coinfected cells (both OP7 and PR8-RKI viruses at an MOI of 10) showed a reduced HA titer (by 0.8 units), a severe reduction in the infectivity of the released virions (∼3 orders of magnitude), and an overproportional quantity of S7 vRNA in relation to S5 and S8 (intracellularly and in the released virus particles). The lower impact of OP7-4 virus may be explained by the additional point mutation found on the vRNA of S7 in comparison to other OP7 viruses (Fig. 7A) or by smaller amounts of OP7 virions in the OP7-4 working seed.

**FIG 12:**
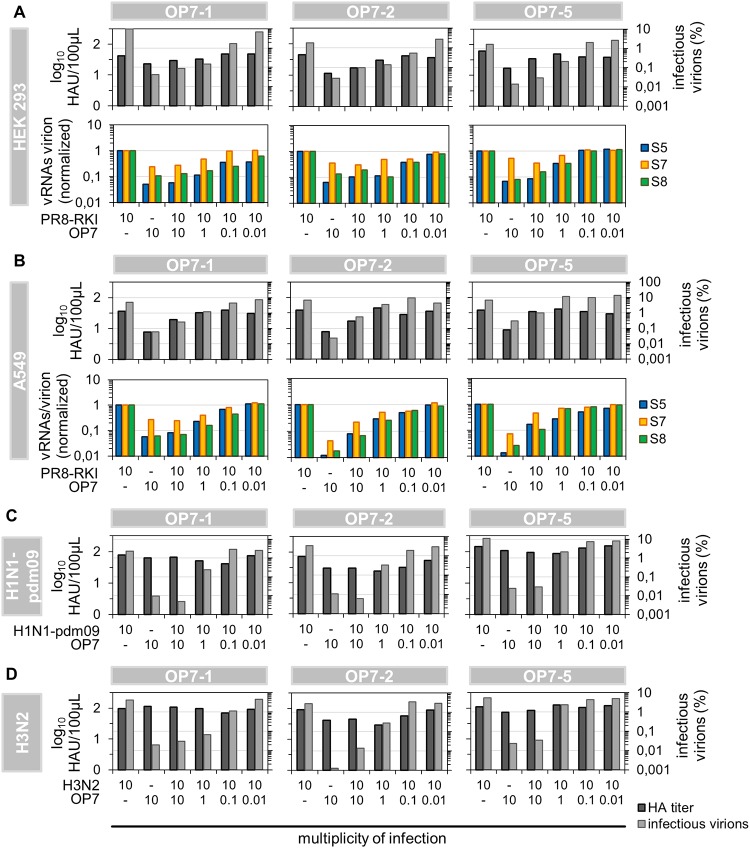
Interference of OP7 virus with replication of various IAV strains in different cell lines. Cells infected with WT virus at an MOI of 10 were simultaneously coinfected with OP7 seed virus at the indicated MOIs until 12 hpi. (A and B) Coinfection of PR8-RKI-infected human HEK 293 (A) and A549 (B) cell lines with OP7 seed virus. (C and D) Interference of OP7 virus with H1N1-pdm09 (C) and H3N2 (D) virus replication in MDCK cells. Infectious virus titers were quantified by a plaque assay, and intracellular and purified vRNAs from virions were quantified by real-time RT-qPCR. Data were used to calculate fractions of infectious virus and numbers of vRNAs per virion using the virus particle concentration (derived from the HA titer). Normalization of vRNAs per virion was based on PR8-RKI virus (as a reference). Three independent infection experiments each were conducted, each using the WT and one OP7 seed virus.

To test whether OP7 virus also shows interference with PR8-RKI virus replication in human cell lines, we next used human embryonic kidney 293 (HEK 293) cells and A549 cells (derived from human lung carcinoma) in coinfection studies. Again, experiments revealed interference, as indicated by the reduction in the HA titer, a strong decrease in the infectivity of released virions, and an overproportional level of S7 vRNA in the produced virus particles compared to cells infected with only PR8-RKI (Fig. 12A and B). Similarly, coinfection studies in MDCK cells also demonstrated interference with the pandemic influenza virus A/California/7/2009 of the H1N1 subtype (H1N1-pdm09) and even with the H3N2 subtype influenza virus A/Hong Kong/4801/2014 (Fig. 12C and D). Taken together, our experiments demonstrated strong interference of OP7 virus with replication of PR8-RKI virus in both MDCK cells and two human cell lines as well as interference with H1N1-pdm09 and H3N2 virus replication.

## DISCUSSION

So far, DIPs have been primarily identified and characterized regarding their large genomic deletions. In contrast, in the present study, we report a yet-unrecognized type of IAV-derived DIP that contains nucleotide substitutions in one of its genome segments. OP7 virus shares very similar features with conventional DIPs, i.e., (i) enhanced genomic replication of the DI genome over other segments, (ii) its predominant packaging into progeny virions, (iii) virus particles that appear to be noninfectious due to the lack of genomic information, and (iv) interference with replication of STV. Yet some of the underlying principles that lead to the above-mentioned observations appear to be different for OP7 virions in comparison to conventional DIPs.

Single-cell infection experiments allowed us to recognize the unusual OP7 phenotype in a subpopulation of cells. In order to multiply the OP7 virions released from these single cells, we infected ∼1 × 10^6^ cells with the corresponding single-cell supernatants, which can result only in a low-MOI scenario, as virus titers of single IAV-infected cells reach only up to roughly 1,000 PFU (16). Typically, low-MOI experiments with virus seeds containing conventional DIPs lead to high infectious virus titers (26), which was also true for the resulting OP7 seed viruses (Fig. 4A). These infection conditions reduce coinfection events, and cells are mostly infected by single virus particles. Hence, STV-infected cells release predominantly infectious viral progeny; however, DIP-only-infected cells cannot contribute to virus production. Yet for a certain time window, these cells may still become coinfected with newly released STV, which, in turn, converts these cells to a (primarily) DIP-producing form (27). Hence, for low MOIs, infectious virions usually dominate in the released virus population. In contrast, using the resulting OP7 seed viruses at high MOIs, we observed a very low infectivity of the released virions (Fig. 4B), an outcome which is also to be expected from seed viruses containing conventional DIPs (26). This infection condition fosters coinfection events and, thus, the complementation of DIP-infected cells with STVs early on. Hence, as a result of the propagation advantage of the DI genomes, mainly noninfectious DIPs accumulate in the resulting virus population.

Our data clearly suggest that OP7 virions are noninfectious due to their reduced vRNA content (Fig. 4B). More specifically, the calculated number of vRNAs per virion was decreased by roughly 1 order of magnitude compared to WT virions, while the quantity of S7 was not affected. These numbers can, theoretically, result in virus populations where (i) only ∼10% of the particles are complete, i.e., they contain each of the eight different genome segments, while the remaining virions contain only S7, or (ii) all virus particles contain S7, yet they lack a large proportion of the remaining seven segments. However, as the fraction of infectious virions was reduced by more than 2 orders of magnitude (compared to WT virions), only the second scenario seems to be conclusive. The remaining infectivity can be explained by the presence of residual STVs, the random packaging of eight functional segments (see below for more details), or the complementation of infected cells with all functional genome segments through coinfection. Furthermore, our conclusion that OP7 virions are defective in virus replication is further supported by the results of plaque purification from PR8-NIBSC virus (which contains OP7 virus). Each plaque is supposed to originate from the infection of a cell by a single virus particle. Yet after 3-fold purification, none of the resultant 43 virus isolates showed the OP7 virus phenotype in infection experiments, which further emphasizes that OP7 virions are propagation incompetent. The origin of the defect in virus replication of OP7 virions differs from that of conventional DIPs, which possess heavy deletions in their DI genome, whereas OP7 virions lack complete genomic vRNA segments, except for their DI genome (i.e., vRNA of S7-OP7).

The segment-specific genome packaging signal sequences of S7 (22) were affected by 17 nucleotide substitutions, which may explain the unusual vRNA content of OP7 virions. Typically, virus assembly and budding are well-organized processes in which the eight different vRNAs are selectively incorporated into each virus particle (28, 29), with the packaging signals being involved (30, 31), although, depending on the strain, up to 20% of virions can still fail to package at least one vRNA (32), which mainly represent the so-called semi-infectious (SI) particles (33). Nevertheless, it was suggested that S7 plays a key role in the IAV genome packaging process, as already only four point mutations in the signal sequence can disrupt vRNA packaging (34). Similar to our results of infection experiments using OP7 seed viruses, the authors of that study observed a dramatic decrease in the infectivity of released virions (of more than 2 orders of magnitude) compared to WT virus replication. This decrease equaled the reduction predicted for a purely random packaging process (35, 36) in which only a minority of virions would incorporate the complete genome. In contrast, Hutchinson and colleagues did not observe an overrepresentation of S7 vRNA in the released virus particles (34). It is conceivable that a disrupted genome packaging mechanism, in combination with the overproportional intracellular quantity of S7-OP7 vRNA, can result in a predominant incorporation of S7-OP7. Alternatively, additional mechanisms may act via the mutated vRNA, which might involve (i) the recently proposed incorporation signal (NCRs at both vRNA ends) and/or (ii) a bundling signal (both terminal coding regions) (37) or even (iii) a selective decrease in the packaging of individual genome segments originating from mutations on another vRNA (38). Extensive research will be required to elucidate the precise mechanisms, e.g., by utilizing reverse genetics (i.e., an eight-plasmid DNA transfection system [39]), which is the subject of ongoing studies. However, the final outcome is a predominant incorporation of S7-OP7 vRNA over other genome segments, an observation that may show similarities to conventional DI RNAs, which are preferentially packaged over their FL counterparts (12, 15).

Previously, artificial IAVs carrying the G3A/C8U superpromoter on the vRNA of either S2 or S3 were reconstituted (20). Upon infection, the observed phenotype showed very similar intracellular features, with respect to viral RNA and protein synthesis from the segments bearing G3A/C8U, compared to S7-OP7 (which carries G3A/C8U) upon OP7 seed virus infection. More specifically, those authors observed (i) a strong decrease in vRNA levels of all genome segments, except for the vRNA carrying G3A/C8U; (ii) an enhanced synthesis of mRNA, cRNA, and protein derived from said segment; and (iii) increased levels of type I IFN in comparison to WT virus replication. Regarding the latter observation, it was shown that this increased induction was likely caused by elevated amounts of immunostimulatory RNA molecules (20). Such an enhanced induction of innate immunity is also observed for infections with conventional DIPs (40–42). Note that the additional G4U substitution (observed in S7-OP7) can also affect promoter function (21). Yet due to the typically dramatic effect of G3A/C8U on viral RNA synthesis (also described elsewhere [e.g., see references 43 and 44]), we conclude that a major part of the altered intracellular viral RNA dynamics upon OP7 seed virus infection must be accounted for by the promoter mutation G3A/C8U found on the vRNA of S7-OP7.

Importantly, note that the G3A/C8U mutation alone does not appear to result in the whole OP7 virus phenotype, as vRNA segments bearing G3A/C8U were not predominantly packaged into progeny virions in the context of an infection (20), unlike S7-OP7 in OP7 virus infection. This indicates that additional mutations (found on S7-OP7) are necessary, beyond G3A/C8U, for the defective and interfering phenotype of OP7 virus. Moreover, it was not described that the G3A/C8U mutation results in a DIP-like phenotype (20, 43–45). The G3A/C8U substitutions have so far been only artificially introduced into the vRNA of IAVs (20, 43–45). It is thus remarkable that S7-OP7 seemed to have obtained G3A/C8U “naturally” by selection. As a result, the genomic vRNA of S7-OP7 accumulates to roughly 10-fold-higher intracellular levels than other genome segments. This feature again shows similarities to conventional DI genomes, which are preferentially synthesized over their FL counterparts (11, 12) but for another reason, i.e., presumably as a result of faster accumulation due to their reduced length (2, 13, 14).

The coding region of S7-OP7 showed 33 point mutations, resulting in two nonconservative amino acid substitutions for both the M1 and M2 (ion channel) proteins. Among other functions, these proteins are also important for virus assembly (46), which may provide an additional explanation for the irregular vRNA content of OP7 virions. Moreover, alterations in the proteins can also affect virus morphology, which show a variety of morphotypes, including filamentous virions (47, 48). Yet OP7 virions appeared similar to WT virus particles but slightly smaller, which may be conclusive owing to their reduced vRNA content. Interestingly, DIPs from vesicular stomatitis virus are smaller as well, due to the reduced size of their DI genomes (49).

The M1 protein is also involved in the nuclear export of vRNPs (25). Although we did not identify alterations in the NLS and NES of M1-OP7, the protein nevertheless showed an unusually high level of accumulation in the nucleus upon OP7 seed virus infection. Concurrently, it appeared that a fraction of vRNPs were retained in the nucleus (compared to WT virus replication). In this context, M1 is thought to mediate the binding of the viral nuclear export protein (NEP) and the vRNPs, which in turn form a complex that is exported from the nucleus by the NES located on NEP (25). Conceivably, the sites of binding of M1-OP7 to NEP and/or the vRNPs are perturbed, which leads to the nuclear retainment of both M1-OP7 and vRNPs. Yet it must be assumed that some functional M1 protein is still synthesized from the coinfecting STV, which may explain the marked nuclear export of vRNPs at early times postinfection. The perturbed function of the mutated M1-OP7 may contribute to the interfering ability of OP7 virus or even to its defect in virus replication. Reconstitution of pure OP7 virus seeds using reverse genetics (39), in combination with overexpression experiments involving M1 or M1-OP7 (and other viral proteins) in OP7 or WT virus-infected cells, respectively, is the subject of ongoing studies and might shed more light into the role and functionality of M1-OP7.

OP7 virions may be a promising candidate for antiviral therapy, as they show strong interference with virus replication of relevant IAV strains and interference in human cell lines. Furthermore, the enhanced induction of innate immunity observed upon OP7 seed virus infection may be further beneficial in the context of antiviral therapy. More precisely, the same stimulation, also induced by infection with conventional DIPs (40–42), is regarded as being useful for potential panspecific treatment of respiratory virus diseases utilizing conventional DIPs (4). The appropriateness of OP7 virions for antiviral therapy may be further investigated *in vivo*, e.g., in mice or in ferrets, as was previously accomplished using conventional DIPs (50–52).

Interestingly, the presence of OP7 virus was presumably noticed previously, yet it was not recognized that it constituted separate virus particles, in particular a separate form of DIP. More specifically, the molar ratio of S7 vRNA relative to other genome segments was increased in virus preparations containing conventional IAV-derived DIPs (12, 53–55). Note that we made a similar observation for virus populations with increased numbers of OP7 virions (Fig. 4B). It was presumed either that some DIPs contain only S7 or that they are polyploid with respect to S7 vRNA (54). However, the presence of potential point mutations was not investigated; rather, the only indications of DIPs were large deletions in some vRNAs. Thus, OP7 virions may have been overlooked for IAVs, in particular as a distinct type of DIP with nucleotide substitutions in its genome. It is conceivable that similar DI genomes also exist in other IAV preparations, which have not been recognized so far, as DIPs are traditionally primarily studied based on their large genomic deletions. Yet awareness of the potential presence of such DIPs may be important regarding the interpretation of experimental results, as they exert strong effects, both on virus replication and on the host cell response, similarly to conventional DIPs (1–3, 40–42, 56, 57).

## MATERIALS AND METHODS

### Cells and viruses.

MDCK cells (catalog number 84121903; ECACC) were cultivated in Glasgow minimum essential medium (GMEM) with 10% fetal bovine serum (FBS) and 1% peptone. HEK 293 (ATCC CRL-1573) and A549 (ATCC CCL-185) cell lines were maintained in Dulbecco’s modified Eagle’s medium (DMEM) containing 10% FBS. All cultivations and infections were performed at 37°C in a 5% CO_2_ atmosphere. Infection medium was prepared by adding porcine trypsin to a final concentration of 5 *N*α-benzoyl-l-arginine ethyl ester (BAEE) U/ml to the corresponding serum-free medium. Influenza virus strain PR8 was provided by the RKI (catalog number 3138) and the NIBSC (catalog number 99/716). Strains H3N2 (catalog number 15/192) and H1N1-pdm09 (catalog number 10/122) were supplied by the NIBSC. Seed virus titers were determined by a standard TCID_50_ assay using MDCK cells (58), and MOIs were based on this titer.

### Isolation of single infected cells.

Isolations were performed as described previously (16). In brief, confluent MDCK cells in 9.6-cm^2^ dishes were infected at the indicated MOIs in 250 μl of infection medium. During the first hour of incubation, the dish was rocked. The medium volume was then increased to 2 ml, and cells were incubated for another 1.5 h. After washing (twice) with phosphate-buffered saline (PBS), cells were trypsinized for 10 to 15 min. Trypsinization was stopped using cell maintenance medium (containing 10% FBS). The homogenized cell suspension was serially diluted in prewarmed (37°C) infection medium. Subsequently, 50 μl of the diluted cell suspension (concentration of 1 cell per 50 μl) was quickly added to each well of a prewarmed 384-well plate (catalog number 781901; Greiner) using an electronic multichannel/multistep pipette. Plates were incubated until 12 hpi. After brief centrifugation at 150 × *g*, we identified individual wells containing single cells by phase-contrast microscopy. Supernatants were immediately subjected to plaque assays to quantify virus yields. The remaining single cells were washed with PBS, and 5 μl of a diluted bovine serum albumin (BSA) solution (catalog number B14; Thermo Scientific) at a concentration of 1 mg/ml was added to the wells. The 384-well plate was sealed and immediately stored at −80°C until use for real-time RT-PCR.

### Plaque assay.

Complete supernatants of infected single cells were investigated for the virus titer (PFU per cell) using two dilutions (either 90% or 10% of the total sample). For cell population-based samples, we prepared serial 10-fold dilutions. A total of 250 μl of each dilution was incubated on MDCK cells in 6-well plates for 1 h. During incubation, the plate was rocked. After removal of the supernatant, cells were overlaid with 1% agar (in infection medium) and incubated for 4 days. Cells were then fixed with methanol and stained using a 0.2% crystal violet solution. The plaque count was determined using light microscopy.

### Cell population-based infection.

Confluent cells in 9.6-cm^2^ dishes were infected at the indicated MOIs in 250 μl of infection medium. During 1 h of incubation, the dish was rocked. The inoculum was removed, cells were washed twice with PBS, and 2 ml of infection medium was added. For each investigated time point postinfection, one dish was sampled.

Aliquots of supernatants were stored at −80°C until virus titration or the purification of vRNA in the released virions using a NucleoSpin RNA virus kit (Macherey-Nagel) according to the manufacturer’s instructions. The remaining cells were then washed twice with PBS. Lysis of cells and intracellular RNA extraction were performed using a NucleoSpin RNA kit (Macherey-Nagel). Purified vRNAs from virus particles and intracellular vRNA, mRNA, and cRNA were quantified by real-time RT-qPCR. The viral RNA levels per cell were calculated based on the cell count at the time point of infection.

Fractions of infectious virus particles and quantities of vRNA per virion were calculated based on the virus particle concentrations, derived from HA titers. For PR8-RKI virus, we obtained a vRNA copy number per virus particle of roughly 5 for S5, S7, and S8, which is of the same order of magnitude and thus in reasonable agreement with the expected value of 1. Hence, numbers of vRNAs per virion of PR8-RKI virus were used for normalization for all remaining viruses (as a reference).

### Virus quantification.

Virus titers of cell population-based infections were determined based on a standard TCID_50_ assay using MDCK cells (58) and an HA assay (59). HA titers were expressed as log_10_ HA units per test volume (log_10_ hemagglutinating units [HAU]/100 μl). Virus particle concentrations (*c*_virus_) (virions per milliliter) were calculated, assuming that agglutination occurs up to a dilution in which the number of virions equals the number of erythrocytes (60). Thus, the calculation was based on the HA titer and the cell concentration of the erythrocyte suspension (2 × 10^7^ cells/ml).

$$c_{virus}=2\;\times {\;10}^{7}\times {\;10}^{\left({log}_{10}\;HAU/100\;\;\mu l\right)}$$

### Real-time RT-qPCR.

Real-time RT-qPCR was utilized for absolute quantification of (i) intracellular vRNA of single-cell samples; (ii) intracellular vRNA, mRNA, and cRNA of cell population-derived samples; and (iii) purified vRNA from virus particles. For this, we derived a primer combination from a previously reported method (61) that enables polarity- and gene-specific amplification of individual IAV RNAs. A tagged primer (Table 1) was used for RT, and qPCR primers are listed in Table 2. To facilitate absolute quantification, we generated RNA reference standards, and numbers of viral RNAs were calculated based on calibration curves.

**TABLE 1:** Tagged primers for RT (related to real-time RT-qPCR)

Target(s)	RNA type	Primer name	Sequence (5′→3′)
Segment 5	vRNA	S5 tagRT for	ATTTAGGTGACACTATAGAAGCGAGTGATTATGAGGGACGGTTGAT
	cRNA	S5 tagRT rev	GCTAGCTTCAGCTAGGCATC AGTAGAAACAAGGGTATTTTTCTT

Segment 7	vRNA	S7 tagRT for	ATTTAGGTGACACTATAGAAGCGAGCCGAGATCGCACAGAGACTT
	cRNA	S7 tagRT rev	GCTAGCTTCAGCTAGGCATCAGTAGAAACAAGGTAGTTTTTTAC

Segment 8	vRNA	S8 tagRT for	ATTTAGGTGACACTATAGAAGCGGATAGTGGAGCGGATTCTG
	cRNA	S8 tagRT rev	GCTAGCTTCAGCTAGGCATC AGTAGAAACAAGGGTGTTTTTTAG

Segments 5, 7, and 8	mRNA	Oligo tagdTRT	GTAAAACGACGGCCAGTTTTTTTTTTTTTTTTT

**TABLE 2:** Primers for qPCR (related to real-time RT-qPCR)

Target	RNA type(s)	Primer name	Sequence (5′→3′)
Introduced tag sequence	vRNA	vRNA tagRealtime for	ATTTAGGTGACACTATAGAAGCG
	cRNA	cRNA tagRealtime rev	GCTAGCTTCAGCTAGGCATC
	mRNA	mRNA tagRealtime rev	GTAAAACGACGGCCAGT

Segment 5	vRNA	Seg 5 Realtime rev	CGCACTGGGATGTTCTTC
	cRNA and mRNA	Seg 5 Realtime for	GGAAAGTGCAAGACCAGAAGAT

Segment 7	vRNA	Seg 7 Realtime rev	TGAGCGTGAACACAAATCCTAAAA
	cRNA and mRNA	Seg 7 Realtime for	CATTGGGATCTTGCACTTGACATT

Segment 8	vRNA	Seg 8 Realtime rev	CACTTTCTGCTTGGGTATGA
	cRNA and mRNA	Seg 8 Realtime for	GGCGGGAACAATTAGGTCAGA

For *in vitro* synthesis of the reference standards, we used plasmids carrying the complete sequences of vRNA, mRNA, and cRNA (of the corresponding segments) by conventional PCR using Phusion high-fidelity DNA polymerase (Thermo Scientific) according to the manufacturer’s instructions. Thereby, the primers (Table 3) introduced a T7 promoter sequence (in the desired orientation) into the PCR products. After purification (InnuPrep PCRpure kit; Analytik Jena), we used the PCR products for *in vitro* transcription (TranscriptAid T7 high-yield transcription kit; Thermo Scientific). Final purification of the RNA reference standards was conducted using a NucleoSpin RNA cleanup kit (Macherey-Nagel), and standards were stored at −80°C until use.

**TABLE 3:** Primers for RNA reference standard generation (related to real-time RT-qPCR)

Target	RNA type	Primer name	Sequence (5′→3′)
Segment 5	cRNA	S5 Uni T7 for	TAATACGACTCACTATAGGGAGCAAAAGCAGGGTAGATAATC
		S5 Uni rev	AGTAGAAACAAGGGTATTTTTC
	vRNA	S5 Uni for	AGCAAAAGCAGGGTAGATAATC
		S5 Uni T7 rev	TAATACGACTCACTATAGGGAGTAGAAACAAGGGTATTTTTC
	mRNA	S5 Uni T7 for	TAATACGACTCACTATAGGGAGCAAAAGCAGGGTAGATAATC
		S5 dT rev	TTTTTTTTTTTTTTTTCTTTAATTGTC

Segment 7	cRNA	S7 Uni T7 for	TAATACGACTCACTATAGGGAAGCGAAAGCAGGTAG
		S7 Uni rev	AGTAGAAACAAGGTAGTTTTT
	vRNA	S7 Uni for	AGCGAAAGCAGGTAG
		S7 Uni T7 rev	TAATACGACTCACTATAGGGAAGTAGAAACAAGGTAGTTTTT
	mRNA	S7 Uni T7 for	TAATACGACTCACTATAGGGAAGCGAAAGCAGGTAG
		S7 dT rev	TTTTTTTTTTTTTTTTACTCCAGCTCT

Segment 8	cRNA	S8 Uni T7 for	TAATACGACTCACTATAGGGAGAAAAAGCAGGGTGACAAA
		S8 Uni rev	AGTAGAAACAAGGGTGTTTT
	vRNA	S8 Uni for	AGAAAAAGCAGGGTGACAAA
		S8 Uni T7 rev	TAATACGACTCACTATAGGGAAGTAGAAACAAGGGTGTTTT
	mRNA	S8 Uni T7 for	TAATACGACTCACTATAGGGAGAAAAAGCAGGGTGACAAA
		S8 dT rev	TTTTTTTTTTTTTTTTAGTACTAAATAAGCTGAAACGAG

For RT, we mixed 1 μl of the RNA sample with 0.5 μl of deoxynucleoside triphosphates (dNTPs) (10 mM) and 0.5 μl of the RT primer (10 μM for the mRNA primer or 1 μM for the vRNA and cRNA primers) and filled up to a volume of 6.5 μl with nuclease-free water. Incubation was performed at 65°C for 5 min and then for 5 min at different temperatures: 42°C for mRNA or 55°C for vRNA and cRNA measurements. During the latter step, we added a prewarmed mixture (42°C for mRNA or 55°C for vRNA and cRNA measurements) consisting of 2 μl 5× RT buffer, 0.25 μl (50 U) Maxima H Minus reverse transcriptase, and 1.25 μl nuclease-free water (all reagents from Thermo Scientific). RT was conducted for 30 min at 60°C, followed by termination at 85°C for 5 min. In addition, we reverse transcribed RNA reference standards in 10-fold dilution steps from 1 to 1 × 10^−7 ^ng. Each reaction mixture contained (optionally) cellular total RNA (to conform with intracellular RNA samples): (i) 350 fg for single-cell-based measurements, (ii) 350 ng for population-based measurements, and (iii) no total RNA for measurements of vRNA from purified virions. The cDNA reaction products were then diluted to 20 μl in nuclease-free water and stored at −20°C or immediately subjected to qPCR analysis.

For qPCR, we used the Rotor-Gene Q real-time PCR cycler (Qiagen). The qPCR mix (10 μl) contained 1× Rotor-Gene SYBR green PCR mix (Qiagen), 500 nM each primer, and 3 μl of diluted cDNA. An initial denaturation step was conducted at 95°C for 5 min, followed by 40 PCR cycles (two-step protocol) of 95°C for 10 s and 62°C for 20 s. Afterwards, melting-curve analysis was performed from 65°C to 90°C.

### Absolute quantification of viral RNAs.

To calculate absolute quantities of viral RNAs, we plotted the *C_T_* (threshold cycle) values (from qPCR) of the serially 10-fold-diluted RNA reference standards (ordinate) against the log_10_ number of RNA molecules, *n*_molecules_ (abscissa), to generate calibration curves (linear regression). *n*_molecules_ was calculated based on the quantity of the standard, *m*_STD_ (nanograms); the fragment length, *N*_bases_ (base pairs); the average mass of 1 base [*k* = 340 (daltons/base pair)]; and the Avogadro constant, *N*_A_ (per mole).

$$n_{molecules}\;=\;\frac{m_{STD}}{N_{bases}\;\times \;k\;\times {\;N}_{A}^{-1}\;\times \;10^{9}}$$

Using the *C_T_* value of a sample, the number of viral RNA molecules, *Q*_sample_, was calculated by considering the slope (*m*) and *y* intercept (*b*) of the calibration curve; the coefficient of dilution of the RT reaction, *F*_RT_; and the total volume of the RNA sample, *V*_sample_ (microliters).

$$Q_{sample}=10^{\left(\frac{C_{T\;-\;\;b}}{m}\right)}\;\times \;F_{RT}\;\times \;V_{sample}$$

### Segment-specific PCR.

Purified vRNAs of virions, released from infected cells (MOI = 10; 12 hpi), were subjected to RT-PCR for two different purposes: (i) investigation of the presence of subgenomic RNAs and (ii) determination of the vRNA sequence (described in more detail below). For RT, we used a universal “Uni12” primer (62), which hybridizes to the conserved 3′ ends of all eight genome segments, to synthesize all cDNAs in one reaction. In subsequent PCR, we used individual reactions for each segment. The primer sequences (Table 4) comprise the conserved 3′- or 5′-terminal vRNA end in conjunction with a segment-specific portion to allow for the specific amplification of the complete genome segment. Note that for amplification and sequencing of S7-OP7 vRNA, we used adapted primers (Table 4).

**TABLE 4:** Primers related to segment-specific PCR

Reaction	Target	Primer name	Sequence (5′→3′)
RT	All segments (WT)	Uni12	AGCAAAAGCAGG
	Segment 7 (OP7 virus)	S7-OP7 RT	AAGCAGGTAGATATTGAAAG

PCR	Segment 1	S1 Uni for	AGCGAAAGCAGGTCAATTAT
		S1 Uni rev	AGTAGAAACAAGGTCGTTTTTAAAC
	Segment 2	S2 Uni for	AGCGAAAGCAGGCAAACCAT
		S2 Uni rev	AGTAGGAACAAGGCATTTTTTCATG
	Segment 3	S3 Uni for	AGCGAAAGCAGGTACTGATCC
		S3 Uni rev	AGTAGAAACAAGGTACTTTTTTGG
	Segment 4	S4 Uni for	AGCAAAAGCAGGGGAA
		S4 Uni rev	AGTAGAAACAAGGGTGTTTT
	Segment 5	S5 Uni for	AGCAAAAGCAGGGTAGATAATC
		S5 Uni rev	AGTAGAAACAAGGGTATTTTTC
	Segment 6	S6 Uni for	AGCGAAAGCAGGGGTTTAAAATG
		S6 Uni rev	AGTAGAAACAAGGAGTTTTTTGAAC
	Segment 7	S7 Uni for	AGCGAAAGCAGGTAGATATTG
		S7 Uni rev	AGTAGAAACAAGGTAGTTTTTTAC
	Segment 7 (OP7 virus)	S7-OP7 PCR for	AAGCAGGTAGATATTGAAAG
		S7-OP7 PCR rev	AGTAGAAACAAGGTAGTTTT
	Segment 8	S8 Uni for	AGAAAAAGCAGGGTGACAAA
		S8 Uni rev	AGTAGAAACAAGGGTGTTTT

For RT, 10 μl of RNA was mixed with 1 μl dNTPs (10 mM) and 1 μl primer (10 mM) and filled up to a volume of 14.5 μl with nuclease-free water. Incubation was conducted at 65°C for 5 min and at 4°C for 5 min. We then added 4 μl of 5× reaction buffer, 50 U (0.5 μl) RevertAid H Minus reverse transcriptase, 20 U (0.5 μl) RiboLock RNase inhibitor, and 0.5 μl nuclease-free water (all reagents from Thermo Scientific) and incubated the mixture at 42°C for 60 min. RT was terminated at 70°C for 10 min. cDNA was stored at −20°C or immediately subjected to PCR.

For PCR, 2 μl cDNA was combined with 4 μl 5× Phusion HF buffer, 2 μl MgCl_2_ (10 mM), 1 μl dNTPs (10 mM), 1 μl of each primer (10 μM), 0.2 μl (0.4 U) Phusion DNA polymerase, and 8.8 μl nuclease-free water (all reagents from Thermo Scientific). Initial denaturation was performed at 98°C for 3 min, followed by 25 PCR cycles of 98°C for 25 s, 54°C for 45 s, and 72°C for different times: 2 min for S1 to S3, 1.5 min for S4 to S6, and 1 min for S7 and S8. A final elongation step was conducted at 72°C for 10 min. PCR products were then visualized using agarose gel electrophoresis.

### Determination of vRNA sequences.

We determined the sequence of purified vRNA from virions (released upon infection at an MOI of 10 at 12 hpi). For sequencing of the coding regions, we used segment-specific PCR (as described above) to amplify the complete segments. After purification, the PCR products were sequenced using the same PCR primers. All sequencing reactions were conducted by Eurofins Genomics (Ebersberg, Germany), utilizing Sanger sequencing.

For sequencing of the terminal vRNA ends, we derived a modified procedure from a previously reported method (63), which is based on the circularization of vRNA using an RNA ligase. The subsequent amplification of the junction region (containing the vRNA ends) was performed by RT-PCR. For RT, a random hexamer primer was used. In subsequent PCR (primers are listed in Table 5), we used a segment-specific primer in combination with a second primer that was designed across the junction of the 3′ and 5′ vRNA ends. After purification, sequencing was undertaken with the sequencing primers (indicated by x's in Table 5). Note that the identities of the terminal 2 bp of each vRNA end were not determined (due to the primer design).

**TABLE 5:** Primers related to vRNA sequence determination

Target	Primer name	Sequence (5′→3′)	Sequencing primer
3′ end of segment 5	3′ Seq S5 2bp for	GAAAAATACCCTTGTTTCTACTAG	
	3′ Seq S5 rev	AGTTCTCTCATCCACTTTCCGT	x

3′ end of segment 7	3′ Seq S7 2bp for	GTAAAAAACTACCTTGTTTCTACTAG	
	3′ Seq S7 rev	TATGAGACCGATGCTGGGAG	x

3′ end of segment 7 (OP7 virus)	3′ Seq S7-OP7 2bp for	GTAAAAAACTACCTTGTTTCTACTAG	
	3′ Seq S7-OP7 rev	GTCACAGTCCCCATCCTGTT	x

3′ end of segment 8	3′ Seq S8 2bp for	AAAAACACCCTTGTTTCTACTAG	
	3′ Seq S8 rev	TTTATCCATGATCGCCTGGT	x

5′ end of segment 5	5′ Seq S5 for	ACCAATCAACAGAGGGCATC	x
	5′ Seq S5 2bp rev	TGATTATCTACCCTGCTTTCGCTAG	

5′ end of segment 7	5′ Seq S7 for	TAGCTCCAGTGCTGGTCTGA	x
	5′ Seq S7 2bp rev	TTTCAATATCTACCTGCTTTCGCTAG	

5′ end of segment 7 (OP7 virus)	5′ Seq S7-OP7 for	TCCAGTGCTGGTCTGAAAGA	x
	5′ Seq S7-OP7 2bp rev	TCAACATCTACCTGCTTTCACTAG	

5′ end of segment 8	5′ Seq S8 for	TCACCATTGCCTTCTCTTCC	x
	5′ Seq S8 2bp rev	TGTCACCCTGCTTTCGCTAG	

Circularization was performed by mixing 11.5 μl of the RNA sample with 4 μl (40 U) of T4 RNA ligase 1, 2 μl of 10× T4 RNA ligase reaction buffer, 2 μl of a 10 mM ATP solution (all reagents from New England BioLabs), and 0.5 μl (20 U) of RiboLock RNase inhibitor (Thermo Scientific). The mixture was incubated for 1 h at 37°C, followed by heat inactivation at 65°C for 15 min. We immediately reverse transcribed the circularized RNA.

For RT, a reaction mixture containing 4 μl ligated RNA, 1 μl (0.2 μg) random hexamer primer, 1 μl of dNTPs (10 mM), and 8.5 μl of nuclease-free water was incubated at 65°C for 5 min (all reagents from Thermo Scientific) and immediately transferred on ice. We then added 4 μl of 5× RT buffer, 0.5 μl (100 U) Maxima H Minus reverse transcriptase, 0.5 μl (20 U) RiboLock RNase inhibitor, and 0.5 μl of nuclease-free water (all reagents from Thermo Scientific). Incubation was conducted at 25°C for 10 min and then at 50°C for 30 min. Termination was performed at 85°C for 5 min. cDNA was stored at −20°C or immediately subjected to PCR.

The PCR mix consisted of 4.5 μl of the RT product, 6 μl 5× Phusion HF buffer, 3 μl MgCl_2_ (10 mM), 1.5 μl dNTPs (10 mM), 1.5 μl of each primer (10 μM), 0.3 μl (0.6 U) Phusion DNA polymerase, and 11.7 μl of nuclease-free water (all reagents from Thermo Scientific). The cycling conditions comprised an initial denaturation step for 105 s at 98°C and then 40 PCR cycles of 10 s at 98°C, 30 s at 60°C, and 40 s at 72°C. A final elongation step was conducted at 72°C for 10 min. All PCR products were excised from gels (after agarose gel electrophoresis) and then purified using the QIAquick gel extraction kit (Qiagen) according to the manufacturer’s instructions.

### Analysis of innate immune response.

Expression of IFN beta and Mx1 in infected cell populations was assessed using real-time RT-qPCR. For this, 500 ng of purified intracellular RNA was reverse transcribed using an oligo(dT) primer and Maxima H Minus reverse transcriptase (both from Thermo Scientific) according to the manufacturer’s instructions. Subsequently, we performed qPCR using primers listed in Table 6 and a Rotor-Gene Q real-time PCR cycler (Qiagen). The qPCR mix (10 μl) contained 1× Rotor-Gene SYBR green PCR mix (Qiagen), 500 nM each primer, and 3 μl of diluted cDNA. Initial denaturation was conducted at 95°C for 5 min, followed by 40 PCR cycles (two-step protocol) of 95°C for 10 s and 62°C for 20 s. Gene expression was expressed as fold induction (over mock-infected cells) and calculated using the ΔΔ*C_T_* method with 18S rRNA as the reference gene.

**TABLE 6:**
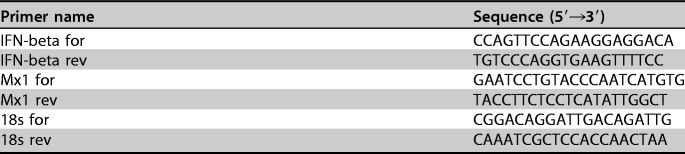
qPCR primers (related to analysis of innate immune response)

### Analysis of intracellular viral proteins.

At the indicated time points, infected MDCK cell populations were washed twice with PBS. We then added 150 μl of radioimmunoprecipitation assay (RIPA) buffer. Cells were harvested using a cell scraper and homogenized using 0.2-μm syringes. After centrifugation (10,000 × *g* for 10 min at 4°C), aliquots of supernatants were stored at −80°C until WB was performed. For WB, we used a polyvinylidene diﬂuoride (PVDF) membrane. Mouse anti-NP monoclonal antibody (mAb) (catalog number ab128193; Abcam) was used at a dilution of 1:2,000, rabbit anti-PA polyclonal antibody (pAb) (catalog number GTX125932; GeneTex) was diluted to 1:10,000, mouse anti-M1 mAb (catalog number MCA401; AbD Serotec) was used at a dilution of 1:1,000, and mouse anti-glyceraldehyde-3-phosphate dehydrogenase (GAPDH) mAb (catalog number CB1001; Merck) was diluted to 1:5,000. Secondary antibody stainings were performed using donkey anti-mouse pAb conjugated with horseradish peroxidase (HRP) (catalog number 715-036-151; Jackson ImmunoResearch) and HRP-conjugated goat anti-rabbit pAb (catalog number 111-035-003; Jackson ImmunoResearch), both at a dilution of 1:10,000. Proteins on the blots were visualized using SuperSignal West Dura extended-duration substrate (Thermo Scientific).

### Electron microscopy.

Virus particles released in cell population-derived infections (MOI = 10; 12 hpi) were inactivated using β-propiolactone and then visualized utilizing ns-TEM. The samples were bound to a glow-discharged carbon foil-covered grid and stained using 1% uranyl acetate. Grids were imaged at room temperature using a CM-120 BioTwin transmission electron microscope (Philips). Images were acquired using a TemCam-F416 CMOS camera (TVIPS).

### Imaging flow cytometric analysis.

At the indicated time points postinfection, we rocked the population of infected MDCK cells to release detached cells into the infection medium. The supernatant was harvested, and detached cells were separated from the supernatant by centrifugation (300 × *g* for 10 min at 4°C). The remaining adherent cells were trypsinized and afterwards combined with the detached cells from the previous step. Cells were then fixed with paraformaldehyde at a final concentration of 1% (30 min at 4°C) and then washed with PBS. Aliquots were stored in 70% ethanol at −20°C until imaging flow cytometric analysis.

Analysis was performed as described previously (64). In brief, cell samples were washed twice with PBS containing 0.1% BSA and 2% glycine, thereby using centrifugation at 300 × *g* for 10 min at 4°C. Samples were then blocked for 30 min at 37°C in PBS containing 1% BSA. After washing, we performed antibody incubations (always at 37°C for 1 h in the dark). Monoclonal mouse anti-NP antibody mAb61A5 (a gift from Fumitaka Momose) was used at a dilution of 1:500. The antibody preferentially binds to NP in the conformation inherent to the vRNP complex (65). Subsequent to washing, the secondary Alexa Fluor 647-conjugated goat anti-mouse pAb (catalog number A21235; Life Technologies) was used at a dilution of 1:500, and cells were then washed two times. Nuclei were visualized by adding DAPI.

For M1 staining, we used a fluorescein isothiocyanate (FITC)-conjugated mouse anti-M1 mAb (catalog number MCA401FX; AbD Serotec) at a dilution of 1:100. After cells were washed, they were resuspended in 1 ml of PBS. We then added 5 μl PureLink RNase A (Life Technologies) for RNA degradation and 0.5 μl of 7-AAD (Merck) for nuclear staining. Incubation was conducted for 30 min at room temperature in the dark. Finally, cells were washed.

ImageStream X Mark II (Amnis, EMD Millipore) was used for acquisition of 10,000 cells per sample (debris and cell doublets were excluded) at a ×60 magnification. The 375- and 642-nm lasers were utilized for excitation of the vRNP/DAPI-stained samples, and the signals from channel 1 (CH1) and channel 5 (CH5) were acquired along with the bright-field (BF) imagery on CH6. For M1/7-AAD-stained cells, we used the 488- and 561-nm excitation lasers, and for detection, we used CH2 and CH5 with BF images on CH6. Single-stained positive controls were used to adjust laser powers and to acquire compensation files.

We used IDEAS software (version 6.1) for image analysis, using only in-focus single cells for analysis. Fractions of vRNPs in the cell nuclei were calculated based on the quantity of the fluorescence signal that was colocalized with the nuclear signal (derived from DAPI). For this, we created masks “nucleus” and “whole_cell” using functions “morphology” on CH1 and “object” on CH6, respectively. New features were generated, termed “intensity_CH5_nucleus” and “intensity_CH5_whole_cell,” by using the feature “intensity” on CH5 within masks “nucleus” and “whole_cell,” respectively. We then created a combined feature, “FI_in_nucleus,” with the following definition: “intensity_CH5_nucleus”/“intensity_CH5_whole_cell.” CH1- and CH5-double-positive cells (of focused, single cells) were plotted on histograms using this feature. The fraction of fluorescence intensity (FI) in the nucleus (percent) was calculated by multiplying mean values of said feature by 100. M1 localization was assessed the same way but under consideration of the corresponding detection channels.

### Statistics.

Sample size computation was not performed, yet every data set presented in this study is derived from at least three independent experiments. Each independent experiment was both performed and measured independently from one another on different days.

### Data availability.

GenBank accession numbers of all vRNA sequences determined in this study are as follows: MH085254 for S5 of PR8-RKI, MH085255 for S7 of PR8-RKI, MH085256 for S8 of PR8-RKI, MH085233 for S5 of OP7-1, MH085234 for S7 of OP7-1, MH085235 for S8 of OP7-1, MH085236 for S5 of OP7-3, MH085237 for S7 of OP7-3, MH085238 for S8 of OP7-3, MH085239 for S5 of OP7-4, MH085240 for S7 of OP7-4, MH085241 for S8 of OP7-4, MH085242 for S5 of OP7-5, MH085243 for S7 of OP7-5, MH085244 for S8 of OP7-5, MH085245 for S5 of PP-1, MH085246 for S7 of PP-1, MH085247 for S8 of PP-1, MH085248 for S5 of PP-5, MH085249 for S7 of PP-5, MH085250 for S8 of PP-5, MH085251 for S5 of PP-6, MH085252 for S7 of PP-6, and MH085253 for S8 of PP-6.
